# Maintenance of community function through compensation breaks down over time in a desert rodent community

**DOI:** 10.1002/ecy.3709

**Published:** 2022-05-12

**Authors:** Renata M. Diaz, S. K. Morgan Ernest

**Affiliations:** ^1^ School of Natural Resources and Environment University of Florida Gainesville Florida USA; ^2^ Department of Wildlife Ecology and Conservation University of Florida Gainesville Florida USA

**Keywords:** community function, compensation, environmental fluctuations, functional redundancy, zero‐sum dynamic

## Abstract

Understanding the ecological processes that maintain community function in systems experiencing species loss, and how these processes change over time, is key to understanding the relationship between community structure and function and predicting how communities may respond to perturbations in the Anthropocene. Using a 30‐year experiment on desert rodents, we show that the impact of species loss on community‐level energy use has changed repeatedly and dramatically over time, due to (1) the addition of new species to the community, and (2) a reduction in functional redundancy among the same set of species. Although strong compensation, initially driven by the dispersal of functionally redundant species to the local community, occurred in this system from 1997 to 2010, since 2010, compensation has broken down due to decreasing functional overlap within the same set of species. Simultaneously, long‐term changes in sitewide community composition due to niche complementarity have decoupled the dynamics of compensation from the overall impact of species loss on community‐level energy use. Shifting, context‐dependent compensatory dynamics, such as those demonstrated here, highlight the importance of explicitly long‐term, metacommunity, and eco‐evolutionary perspectives on the link between species‐level fluctuations and community function in a changing world.

## INTRODUCTION

Determining the extent to which community‐level properties are affected by species loss, and how and why this changes over time, is key for understanding how communities are structured and how community function may respond to future perturbations (Gonzalez & Loreau, 2009). When species are lost from a community, their contributions to community function (e.g., total productivity or resource use) are also directly lost. Community function may be maintained, however, if, in the new community context, species that remain perform similar functions to the species that were lost, and compensate for the decline in function directly caused by species loss, that is, functional redundancy (Ernest & Brown, 2001; Gonzalez & Loreau, 2009; Rosenfeld, 2002; Walker, 1992, 1995). When compensation via functional redundancy occurs among consumers with a common resource base, it is consistent with a zero‐sum competitive dynamic, in which resources not used by one species are readily absorbed by competitors, and any increases in the abundance of one species must come at the direct expense of others (Ernest et al., 2008; van Valen, 1973).

Because the response of system‐level function to species loss is partially determined by the degree of functional redundancy in a community, processes that cause functional redundancy to change over time can have important consequences for the long‐term maintenance of ecosystem function. Colonization events may buffer community function against species loss, if a community gains species that perform similar functions to the species that were lost (Ernest & Brown, 2001; Leibold et al., 2017). The ability of colonization to supply functionally redundant species depends on the species (and traits) present in the broader metacommunity, and on the rate of dispersal supplying appropriate species to local communities (Leibold et al., 2017).

Even without the addition of new species and traits, however, functional redundancy within a consistent set of coexisting species may fluctuate over time. While, in theory, functional redundancy may occur via the special case of complete niche neutrality (e.g., Hubbell, 2001), it may also occur in niche‐structured systems that contain species that share some traits but differ along other niche axes (Thibault et al., 2010). In these systems, if similar, but non‐identical, species respond to environmental change in similar ways, functional overlap can be maintained or even strengthened. However, if niche differences cause species to respond differently to changing conditions, the degree of functional overlap between those species may decline, resulting in a breakdown in compensation (Fetzer et al., 2015; Loreau, 2004). Over time, as metacommunity dynamics and changing environmental conditions modulate functional redundancy within a community, the extent to which community function is robust to species loss, and the strength of zero‐sum competition, may also be dynamic and context dependent.

Despite logical conceptual support, and evidence from experimental microcosms (Fetzer et al., 2015), there is little empirical documentation of how, and through which mechanisms, temporal changes in functional redundancy modulate the effect of species loss on ecosystem function in natural assemblages. Although relatively plentiful, observational data cannot unambiguously detect compensation through functional redundancy, and even short‐term experiments may not be sufficient to capture temporal variation in compensation (Ernest & Brown, 2001; Houlahan et al., 2007). In contrast, long‐term manipulative experiments are uniquely suited to address this question. In long‐term experiments in which key species are removed from a community over an extended period of time, the impact of species loss on community function can be directly quantified by comparing community function between complete and manipulated assemblages. As metacommunity dynamics and environmental conditions shift over time, long‐term monitoring can reveal how these processes contribute to changes in functional redundancy and ecosystem function across different time periods. Due to the financial and logistical resources required to maintain and monitor whole‐community manipulations over long timescales, these experiments are rare in natural systems representative of realistic evolutionary, geographic, and environmental constraints (Hughes et al., 2017).

Here, we use a 30‐year experiment on desert rodents to investigate how shifts in functional redundancy alter the effect of species loss on community function over time. In this study, kangaroo rats (*Dipodomys* spp.), the largest and competitively dominant species in the rodent community, have been removed from a subset of experimental plots to explore how the loss of key species affects community function, measured as community‐level metabolic flux (total energy use, or Etot) or total biomass (Ernest et al., 2020). For systems of consumers with a shared resource base, such as this community of granivorous rodents, Etot reflects the total amount of resources being processed by an assemblage, and total biomass directly reflects standing biomass. Both are important metrics of community function (Ernest & Brown, 2001; Lawton, 1994). Long‐term monitoring of this experiment has documented repeated shifts in the habitat and species composition of this system, resulting in distinct time periods characterized by different habitat conditions and configurations of the rodent community (Christensen et al., 2018). Abrupt reorganization events in community composition occurred in 1997 and in 2010, associated with the establishment and subsequent decline of the pocket mouse *Chaetodipus baileyi*. *C. baileyi* is similar in size, and presumably other traits, to kangaroo rats, and its establishment in 1996–1997 drove a pronounced increase in compensation due to functional redundancy between *C. baileyi* and kangaroo rats (Ernest & Brown, 2001; Thibault et al., 2010). Over the course of this experiment, shifting environmental conditions have caused the habitat at the study site to transition from desert grassland to scrub, driving a shift in baseline rodent community composition away from kangaroo rats and favoring other, smaller, granivores (Brown et al., 1997; Ernest et al., 2008). By making comparisons across these time periods, we explored how shifts in community composition and functional overlap among the same species have contributed to long‐term changes in the effect of species loss on community function.

## METHODS

### The Portal Project

The Portal Project consists of a set of 24 fenced experimental plots located approximately 7 miles (1 mile = 1.6 km) east of Portal, AZ, USA, on unceded land of the Chiricahua Apache. Beginning in 1977, kangaroo rats (*Dipodomys spectabilis*, *D. merriami*, and *D. ordii*) have been experimentally excluded from a subset of these plots (exclosures), while all other rodents are allowed access through small holes cut in the plot fencing. Control plots, with larger holes, are accessible to all rodents, including kangaroo rats. Rodents on all plots are censused via monthly bouts of live‐trapping. Each individual captured is identified to species and weighed. For additional details on the site and methodology of the Portal Project, see Ernest et al. (2020).

### Data

We used data for control and exclosure plots from February 1988 until January 2020. The experimental treatments for some plots have changed over time, and we used the subset of plots that have had the same treatments for the longest period of time (Ernest et al., 2020). Four control plots, and five exclosure plots, met these criteria. In order to achieve a balanced sample, we randomly selected four exclosure plots for analysis. We divided the time series into three time periods defined by major transitions in the rodent community surrounding the establishment and decline of *C. baileyi* (Christensen et al., 2018; Ernest & Brown, 2001). The first time period (February 1988–June 1997) precedes *C. baileyi*'s establishment at the site. We defined *C. baileyi*'s establishment date as the first census period in which *C. baileyi* was captured on all exclosure plots (following Bledsoe & Ernest, 2019). During the second time period (July 1997–January 2010), *C. baileyi* was abundant on both exclosure and control plots. This time period ended with a reorganization event in which *C. balieyi* became scarce sitewide. We used January 2010, the midpoint of the 95% credible interval for the date of this reorganization event as estimated in Christensen et al. (2018), as the end date for this time period. The last time period spans from Feburary 2010 to January 2020. For each individual rodent captured, we estimated the individual‐level metabolic rate using the scaling relationship between individual body mass and metabolic rate *b =* 5.69 × (*m* ^0.75^), where *m* is body mass in grams and *b* is metabolic rate (for details, see White et al., 2004). We calculated treatment and species‐level energy use as the sum of the appropriate individuals’ metabolic rates, and total biomass as the sum of individuals’ body mass measurements.

### Statistical analysis of rodent community energy use and biomass

Here, we describe analyses for energy use. For biomass, we repeated these analyses substituting biomass values for energy use throughout. For all variables, we combined data for all plots within a treatment in each monthly census period and calculated treatment‐level means. This is necessary to calculate compensation, and we treated other variables in the same way to maintain consistency. A provisional plot‐level analysis yielded qualitatively equivalent results (Appendix S1). To measure the overall impact of kangaroo rat removal on Etot, we calculated a total energy ratio as the ratio of treatment‐level Etot for kangaroo‐rat exclosure plots relative to unmanipulated control plots, that is Etot_E_/Etot_C_ where Etot_E_ and Etot_C_ are total energy use on exclosures and controls, respectively (Bledsoe & Ernest, 2019; Thibault et al., 2010). This ratio is distinct from compensation, which we defined as the proportion of the energy made available by kangaroo rat removal taken up via compensatory increases in energy use by small granivores (all granivores other than kangaroo rats: *Baiomys taylori*, *C. baileyi*, *Chaetodipus hispidus*, *Chaetodipus intermedius*, *Chaetodipus penicillatus*, *Perognathus flavus*, *Peromyscus eremicus*, *Peromyscus leucopus*, *Peromyscus maniculatus*, *Reithrodontomys fulvescens*, *Reithrodontomys megalotis*, and *Reithrodontomys montanus*). We calculated this as (SG_E_ − SG_C_)/KR_C_, where SG_E_ and SG_C_ are the amount of energy used by small granivores (SG) on exclosure and control plots, respectively, and KR_C_ is the amount of energy used by kangaroo rats (KR) on control plots (Ernest & Brown, 2001). To compare these variables across time periods, we used generalized least squares models (GLS; the R package *nlme*; Pinheiro et al., 2020) of the form (SG_E_ – SG_C_)/KR_C_ ~ time period, for compensation, and Etot_E_/Etot_C_ ~ time period, for the total energy ratio. We included a continuous‐time autoregressive temporal autocorrelation term to account for temporal autocorrelation between values from monthly census periods within each multi‐year time period (for details of model selection, see Appendix S2). To evaluate change in baseline community composition over time, we calculated the proportion of treatment‐level energy use accounted for by kangaroo rats on control plots in each census period (KR_C_/Etot_C_). Proportional energy use is bounded 0–1 and is therefore not appropriate for GLS, so we compared values across time periods using a binomial generalized linear model (GLM) of the form KR_C_/Etot_C_ ~ time period. Finally, we calculated the proportional energy use accounted for by *C. baileyi* (CB) on exclosure and control plots in each census period (CB_E_/Etot_E_ and CB_C_/Etot_C_, respectively). *C. baileyi* was not present at the site prior to 1996, and we restricted the analysis of *C. baileyi* proportional energy use to the second two time periods. We compared *C. baileyi* proportional energy use over time and across treatments using a binomial GLM of the form CB_E_/Etot_E_ ~ time period + treatment. For all models, we calculated estimated means and 95% confidence or credible intervals for time‐period (and, for *C. baileyi*, treatment) level values, and contrasts between time periods (and, for *C. baileyi*, treatments), using the R package *emmeans* (Lenth, 2021). Analyses were conducted in R 4.0.3 (R Core Team, 2020). Data and code are archived at https://doi.org/10.5281/zenodo.5544361 and https://doi.org/10.5281/zenodo.5539880.

## RESULTS

**FIGURE 1:**
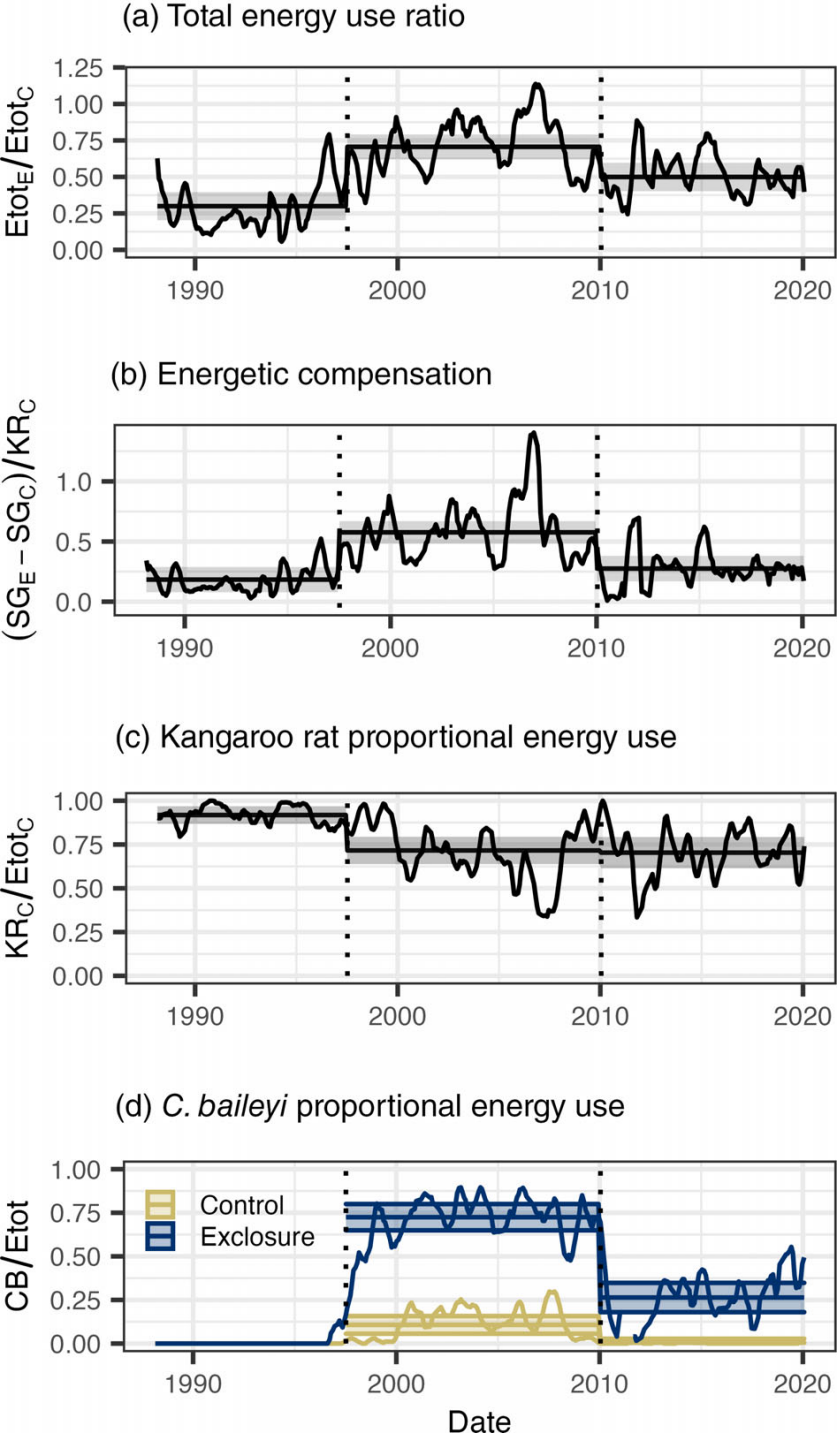
Dynamics of energy use and rodent community composition over time. Lines represent (a) the ratio of total energy use on exclosure plots (Etot_E_) to control plots (Etot_C_), (b) 6‐month moving averages of energetic compensation (calculated as (SG_E_ − SG_C_)/KR_C_, where SG_E_ and SG_C_ are the amount of energy used by small granivores on exclosure and control plots, respectively, and KR_C_ is the amount of energy used by kangaroo rats on control plots), and (c) the share of community energy use accounted for by kangaroo rats on control plots and (d) by *Chaetodipus baileyi* on control (gold) and exclosure (blue) plots. Dotted vertical lines mark the boundaries between time periods used for statistical analysis. Horizontal lines are time‐period estimates from generalized least squares (a, b) and generalized linear (c, d) models, and the semitransparent envelopes mark the 95% confidence or credible intervals

The impact of kangaroo rat removal on community function has changed repeatedly over time, through a combination of abrupt shifts in compensation associated with *C. baileyi*, and long‐term changes in baseline community composition sitewide (Figure 1). These dynamics are qualitatively identical whether function is measured as total energy use (Figure 1; Appendix S2) or total biomass (Appendix S3). The first shift coincided with *C. baileyi*'s establishment in the community beginning in 1996–1997 (Figure 1d). *C. baileyi* rapidly became dominant on exclosure plots and dramatically increased compensation (Figure 1b). From 1997 to 2010, small granivores compensated for an average of 58% of kangaroo rat energy use on control plots (95% interval 48%–67%), an increase from an average of 18% from 1988 to 1997 (95% interval 8%–29%; contrast *p* < 0.001; for complete results of all models, see Appendix S2) from 1997 to 2010. With *C. baileyi*'s addition to the community, the total energy ratio (on exclosures relative to controls; Figure 1a) increased from 30% (20%–40%) to 71% (62%–79%, contrast *p* < 0.014). In the second shift, beginning around 2010, *C. baileyi*'s abundance sitewide dropped precipitously (Figure 1d). *C. baileyi*'s proportional energy use dropped from an average of 72% (65%–80%) to 26% (18%–35%, contrast *p* < 0.001) on exclosure plots, and from 11% (6%–16%) to essentially 0 on control plots (contrast *p* < 0.001). Other species of small granivore did not make compensatory gains to offset the decline in *C. baileyi* (Figure 1b). As a result, compensation declined from an average of 58% (48%–67%) to 28% (17%–38%, contrast *p* = 0.002), a level not significantly different from the 18% (8%–29%, contrast *p* = 0.44) observed prior to *C. baileyi*'s establishment at the site. Somewhat paradoxically, while the total energy ratio also dropped following *C. baileyi*'s decline, from an average of 71% (62%–79%) from 1997 to 2010 to 50% (40%–60%, contrast *p* = 0.0056) from 2010 to 2020, it remained higher than its average of 30% (20%–40%, contrast *p* = 0.0144) from 1988 to 1997 (Figure 1a). Over the course of the experiment, community composition shifted sitewide. In later years, kangaroo rats accounted for a lower proportion of baseline Etot than they did at the beginning of the study (Figure 1c). From 1988 to 1997, kangaroo rats accounted for 92% (87%–97%) of Etot on controls; after 1997, this dropped to an average of approximately 70% (1988–1997 compared to later time periods, both *p* = 0.0004; 1997–2010 and 2020–2020 not significantly different, *p* = 0.976). Because the proportion of Etot directly lost to kangaroo rat removal was smaller from 2010 to 2020 than from 1988 to 1997, the total energy ratio was higher from 2010 to 2020 than it was from 1988 to 1997, even though there was not a detectable difference between the two time periods in the proportion of lost energy being offset through compensation.

## DISCUSSION

The dynamics of rodent community energy use at Portal illustrate that the role of functional redundancy in buffering community function against species loss fluctuates over time, due to changes in both species composition and in the degree of functional overlap among the same species. The 1997 increase in compensation, driven by *C. baileyi*'s establishment at the site, was a clear and compelling instance of colonization from the regional species pool overcoming limitations on functional redundancy (Ernest & Brown, 2001; Leibold et al., 2017). Although the small granivore species originally present in the community did not possess the traits necessary to compensate for kangaroo rats, *C. baileyi* supplied those traits and substantially, but incompletely, restored community function. In contrast, following the community reorganization event in 2010, *C. baileyi* remained present in the community, but ceased to operate as a partial functional replacement for kangaroo rats. This is consistent with fluctuating conditions modulating functional redundancy between similar, but non‐identical, competitors. Kangaroo rats and *C. baileyi* are relatively similar in size and are demonstrably capable of using similar resources. However, *C. baileyi* prefers different, shrubbier microhabitats than kangaroo rats, and the two groups have been observed to replace each other in adjacent habitats (Ernest & Brown, 2001). We suggest that this study site, which has historically been dominated by kangaroo rats, constitutes marginal habitat for *C. baileyi*, and that, while conditions from 1997 to 2010 aligned sufficiently with *C. baileyi*'s requirements to create appreciable functional redundancy between kangaroo rats and *C. baileyi*, conditions since have caused this redundancy to break down. *C. baileyi*'s decline occurred immediately following a period of low plant productivity and low rodent abundance community‐wide, and in the decade following, the site experienced two long and severe droughts (Appendix S4; Christensen et al., 2018). These extreme conditions may themselves have limited *C. baileyi's* fitness at the site, or the community‐wide low abundance event may have temporarily overcome incumbency effects and triggered a community shift tracking longer‐term habitat trends (Christensen et al., 2018; Thibault & Brown, 2008). Regardless of the proximate cause of *C. baileyi*'s decline, the fact that *C. baileyi* remains in the community, but no longer compensates for kangaroo rats, illustrates that changing conditions can have profound effects on community function by modulating the degree of functional redundancy within a consistent set of species.

While changes in compensation have contributed to changes in community function in this system, changes in compensation alone do not fully account for the long‐term changes in the overall impact of kangaroo rat removal on Etot. Since 2010, although the ratio of Etot on exclosure plots relative to control plots declined coinciding with the breakdown in compensation associated with *C. baileyi*, it remained higher than the levels observed prior to 1997 (Figure 1a). This difference between the first and last time periods cannot be explained by an increase in compensation, as compensation from 2010 to 2020 was not greater than pre‐1997 levels (Figure 1b). Rather, the increase in Etot on exclosure plots relative to control plots was the result of a long‐term decrease in the contribution of kangaroo rats to Etot sitewide. Because kangaroo rats accounted for a smaller proportion of Etot on control plots from 2010 to 2020 than they did prior to 1997, their removal had a smaller impact on community function, even though there was not an increase in the degree to which small granivores compensated for their absence. In fact, the comparable levels of compensation achieved in the decades preceding and following *C. baileyi*'s dominance at the site suggest a relatively stable, and limited, degree of functional overlap between kangaroo rats and the original small granivores (i.e., excluding *C. bailyei*). Niche complementarity, combined with changing habitat conditions, may partially explain this phenomenon. It is well‐documented that, while kangaroo rats readily forage in open microhabitats where predation risk can be relatively high, smaller granivores preferentially forage in sheltered microhabitats as an antipredator tactic (Kelt, 2011). Over the course of this experiment, the habitat at this study site has transitioned from an arid grassland to a shrubland (Brown et al., 1997). As sheltered microhabitats became more widespread, small granivores may have gained access to a larger proportion of resources and increased their share of Etot sitewide. However, kangaroo rats may have continued to use resources in open areas, which would have remained inaccessible to smaller granivores even on exclosure plots. The long‐term reduction in the impact of kangaroo rat removal on community function, driven by niche complementarity and consistent niche partitioning, contrasts with the temporary compensatory dynamic driven by functional redundancy with *C. baileyi*. Although changes in the overall effect of species loss are sometimes treated interchangeably with compensation (e.g., Ernest & Brown, 2001 compared to Thibault et al., 2010), it is important to recognize that multiple distinct pathways modulate the long‐term impacts of species loss on community function. Particularly in strongly niche‐structured systems, complementarity effects and fluctuations in functional redundancy may occur simultaneously, with complex and counterintuitive impacts on community function.

Overall, the decadal‐scale changes in energy use among the Portal rodents underscore the importance of long‐term metacommunity dynamics to the maintenance of community function following species loss (see Leibold et al., 2017). Although a single colonization event may allow for temporary compensation via functional redundancy, as conditions shift, species that once compensated may no longer perform that function (see also Isbell et al., 2011). Particularly if limiting similarity prevents similar competitors from specializing on precisely the same habitats (Rosenfeld, 2002), temporary, context‐dependent compensation may be common. To maintain compensation over time, multiple colonization events, supplying species that are functionally redundant under different conditions, may be required. Depending on dispersal rates, and the diversity and composition of regional species pools, this may be unlikely or even impossible. At Portal, dispersal limitation introduced a 20‐year delay in the compensatory response driven by *C. baileyi*. Theoretically, a new species capable of compensating for kangaroo rats, and better suited to conditions at the site since 2010, could restore compensation under present conditions, but it is unclear whether this species exists or if it can disperse to this site. As ecosystems globally undergo reductions in habitat connectivity and regional beta diversity, and enter novel climatic spaces, maintenance of community function via functional redundancy may grow increasingly rare and fragile (Dornelas et al., 2014; Williams & Jackson, 2007).

Finally, the long‐term variability in functional redundancy documented here adds important nuance to our understanding of how zero‐sum dynamics operate in natural assemblages. Theories invoking zero‐sum dynamics, and tests for compensatory dynamics in empirical data, often treat a zero‐sum dynamic as a strong and temporally consistent constraint (Houlahan et al., 2007; Hubbell, 2001). In this framing, any resources made available via species loss should immediately be taken up by other species. This is not consistent with the dynamics that occur at Portal, which has seen extended periods of time when resources are available on exclosure plots but are not used. Rather, these results are more consistent with a zero‐sum constraint operating at metacommunity or evolutionary scales (Leibold et al., 2017; Terry & Rowe, 2015; van Valen, 1973). Over short timescales, or within a closed local assemblage, niche differences may weaken zero‐sum effects, especially under fluctuating conditions. However, over larger temporal and spatial scales, dispersal or evolution may supply new species equipped to use available resources—via either functional redundancy, or niche complementarity allowing them to exploit novel niches. A long‐term, metacommunity, and even macroevolutionary approach may be necessary to fully understand how zero‐sum constraints, functional redundancy, and niche complementarity contribute to the maintenance of community‐level function in the face of species extinctions and changing conditions over time.

## CONFLICT OF INTEREST

The authors declare no conflict of interest.

## Supporting information


Appendix S1

Appendix S2

Appendix S3

Appendix S4

## Data Availability

All data (Diaz 2022a) and code (Diaz 2022b) to reproduce these analyses are archived on Zenodo at https://doi.org/10.5281/zenodo.5544361 and https://doi.org/10.5281/zenodo.5539880.

## References

[ecy3709-bib-0001] Bledsoe, E. K. , and S. K. M. Ernest . 2019. “Temporal Changes in Species Composition Affect a Ubiquitous Species’ Use of Habitat Patches.” Ecology 100(11): e02869. 10.1002/ecy.2869. 31454069 PMC6899716

[ecy3709-bib-0002] Brown, J. H. , T. J. Valone , and C. G. Curtin . 1997. “Reorganization of an Arid Ecosystem in Response to Recent Climate Change.” Proceedings of the National Academy of Sciences USA 94(18): 9729–33. 10.1073/pnas.94.18.9729. PMC23258 11038570

[ecy3709-bib-0003] Christensen, E. M. , D. J. Harris , and S. K. M. Ernest . 2018. “Long‐Term Community Change through Multiple Rapid Transitions in a Desert Rodent Community.” Ecology 99(7): 1523–9. 10.1002/ecy.2373. 29718539

[ecy3709-bib-0032] Diaz, R. 2022a. diazrenata/squareone: v1.0 (v1.0). Zenodo. 10.5281/zenodo.6046666.

[ecy3709-bib-0033] Diaz, R. 2022b. diazrenata/soar: Revisions1 (v1.1). Zenodo. 10.5281/zenodo.5914937.

[ecy3709-bib-0004] Dornelas, M. , N. J. Gotelli , B. McGill , H. Shimadzu , F. Moyes , C. Sievers , and A. E. Magurran . 2014. “Assemblage Time Series Reveal Biodiversity Change but Not Systematic Loss.” Science 344(6181): 296–9. 10.1126/science.1248484. 24744374

[ecy3709-bib-0005] Ernest, S. K. M. , and J. H. Brown . 2001. “Delayed Compensation for Missing Keystone Species by Colonization.” Science 292(5514): 101–4. 10.1126/science.292.5514.101. 11292873

[ecy3709-bib-0006] Ernest, S. K. M. , J. H. Brown , K. M. Thibault , E. P. White , and J. R. Goheen . 2008. “Zero Sum, the Niche, and Metacommunities: Long‐Term Dynamics of Community Assembly.” The American Naturalist 172(6): E257–69. 10.1086/592402. 18947326

[ecy3709-bib-0007] Ernest, S. K. M. , G. M. Yenni , G. Allington , E. K. Bledsoe , E. M. Christensen , R. M. Diaz , K. Geluso , et al. 2020. “The Portal Project: A Long‐Term Study of a Chihuahuan Desert Ecosystem.” BioRxiv, January, 332783. 10.1101/332783.

[ecy3709-bib-0008] Fetzer, I. , K. Johst , R. Schäwe , T. Banitz , H. Harms , and A. Chatzinotas . 2015. “The Extent of Functional Redundancy Changes as Species’ Roles Shift in Different Environments.” Proceedings of the National Academy of Sciences USA 112(48): 14888–93. 10.1073/pnas.1505587112. PMC4672811 26578806

[ecy3709-bib-0009] Gonzalez, A. , and M. Loreau . 2009. “The Causes and Consequences of Compensatory Dynamics in Ecological Communities.” Annual Review of Ecology, Evolution, and Systematics 40(1): 393–414. 10.1146/annurev.ecolsys.39.110707.173349.

[ecy3709-bib-0010] Houlahan, J. E. , D. J. Currie , K. Cottenie , G. S. Cumming , S. K. M. Ernest , C. S. Findlay , S. D. Fuhlendorf , et al. 2007. “Compensatory Dynamics Are Rare in Natural Ecological Communities.” Proceedings of the National Academy of Sciences 104(9): 3273–7. https://www.srs.fs.usda.gov/pubs/29712. 10.1073/pnas.0603798104 PMC1805590 17360637

[ecy3709-bib-0011] Hubbell, S. P. 2001. The Unified Neutral Theory of Biodiversity and Biogeography (MPB‐32). Princeton, NJ: Princeton University Press. https://www.jstor.org/stable/j.ctt7rj8w.

[ecy3709-bib-0012] Hughes, B. B. , R. Beas‐Luna , A. K. Barner , K. Brewitt , D. R. Brumbaugh , E. B. Cerny‐Chipman , S. L. Close , et al. 2017. “Long‐Term Studies Contribute Disproportionately to Ecology and Policy.” Bioscience 67(3): 271–81. 10.1093/biosci/biw185.

[ecy3709-bib-0013] Isbell, F. , V. Calcagno , A. Hector , J. Connolly , W. S. Harpole , P. B. Reich , M. Scherer‐Lorenzen , et al. 2011. “High Plant Diversity Is Needed to Maintain Ecosystem Services.” Nature 477(7363): 199–202. 10.1038/nature10282. 21832994

[ecy3709-bib-0014] Kelt, D. A. 2011. “Comparative Ecology of Desert Small Mammals: A Selective Review of the Past 30 Years.” Journal of Mammalogy 92(6): 1158–78. 10.1644/10-MAMM-S-238.1.

[ecy3709-bib-0016] Lawton, J. H. 1994. “What Do Species Do in Ecosystems?” Oikos 71(3): 367–74. 10.2307/3545824.

[ecy3709-bib-0017] Leibold, M. A. , J. M. Chase , and S. K. M. Ernest . 2017. “Community Assembly and the Functioning of Ecosystems: How Metacommunity Processes Alter Ecosystems Attributes.” Ecology 98(4): 909–19. 10.1002/ecy.1697. 27984663

[ecy3709-bib-0018] Lenth, R. V . 2021. “Emmeans: Estimated Marginal Means, Aka Least‐Squares Means.” https://CRAN.R-project.org/package=emmeans.

[ecy3709-bib-0019] Loreau, M. 2004. “Does Functional Redundancy Exist?” Oikos 104(3): 606–11. 10.1111/j.0030-1299.2004.12685.x.

[ecy3709-bib-0021] Pinheiro, J. , D. Bates , S. DebRoy , D. Sarkar , and R Core Team . 2020. “Nlme: Linear and Nonlinear Mixed Effects Models.” https://CRAN.R-project.org/package=nlme.

[ecy3709-bib-0022] R Core Team . 2020. R: A Language and Environment for Statistical Computing. Vienna: R Foundation for Statistical Computing. https://www.R-project.org/.

[ecy3709-bib-0023] Rosenfeld, J. S. 2002. “Functional Redundancy in Ecology and Conservation.” Oikos 98(1): 156–62. 10.1034/j.1600-0706.2002.980116.x.

[ecy3709-bib-0024] Terry, R. C. , and R. J. Rowe . 2015. “Energy Flow and Functional Compensation in Great Basin Small Mammals under Natural and Anthropogenic Environmental Change.” Proceedings of the National Academy of Sciences 112(31): 9656–61. 10.1073/pnas.1424315112. PMC4534246 26170294

[ecy3709-bib-0025] Thibault, K. M. , and J. H. Brown . 2008. “Impact of an Extreme Climatic Event on Community Assembly.” Proceedings of the National Academy of Sciences USA 105(9): 3410–5. 10.1073/pnas.0712282105. PMC2265133 18303115

[ecy3709-bib-0026] Thibault, K. M. , S. K. M. Ernest , and J. H. Brown . 2010. “Redundant or Complementary? Impact of a Colonizing Species on Community Structure and Function.” Oikos 119(11): 1719–26.

[ecy3709-bib-0027] van Valen, L. 1973. “A New Evolutionary Law.” Evolutionary Theory 1(1): 1–30.

[ecy3709-bib-0028] Walker, B. 1995. “Conserving Biological Diversity through Ecosystem Resilience.” Conservation Biology 9(4): 747–52. 10.1046/j.1523-1739.1995.09040747.x.

[ecy3709-bib-0029] Walker, B. H. 1992. “Biodiversity and Ecological Redundancy.” Conservation Biology 6(1): 18–23. 10.1046/j.1523-1739.1992.610018.x.

[ecy3709-bib-0030] White, E. P. , S. K. M. Ernest , and K. M. Thibault . 2004. “Trade‐Offs in Community Properties through Time in a Desert Rodent Community.” The American Naturalist 164(5): 670–6. 10.1086/424766. 15540156

[ecy3709-bib-0031] Williams, J. W. , and S. T. Jackson . 2007. “Novel Climates, no‐Analog Communities, and Ecological Surprises.” Frontiers in Ecology and the Environment 5(9): 475–82. 10.1890/070037.

