# Supplementary material for: Maintenance of community function through compensation breaks down over time in a desert rodent community
Source: Ecology. 2022 May 12;103(7):e3709. doi: 10.1002/ecy.3709 (PMC9287087; doi:10.1002/ecy.3709)
Supplement: Supplementary file 1 — Appendix S1 [file ECY-103-0-s002.pdf]

Appendix S1 - Plot-level analysis

Supplemental information for "Maintenance of community function through compensation breaks down over time in a desert rodent community", by Renata M. Diaz and S. K. Morgan Ernest, in *Ecology.*

Fully annotated code and RMarkdown documents to reproduce these analyses are available at https://doi.org/10.5281/zenodo.5544361 and https://doi.org/10.5281/zenodo.5539880.

Table of Contents

Explanation 3

Compensation 4

Model specification and selection 4

Table S1. Model comparison for compensation 4

Results 4

Table S2. Coefficients from linear mixed-effects model for compensation 4

Table S3. Estimates from linear mixed-effects model for compensation 4

Table S4. Contrasts from linear mixed-effects model for compensation 5

Total energy use 6

Model specification and selection 6

Table S5. Model comparison for total energy ratio. 6

Results 6

Table S6. Coefficients from linear mixed-effects model on total energy ratio 6

Table S7. Estimates from linear mixed-effects model on total energy ratio 6

Table S8. Contrasts from linear mixed-effects model on total energy ratio 7

Kangaroo rat proportional energy use 8

Model specification and selection 8

Table S9. Model comparison for Dipodomys proportional energy use. 8

Results 8

Table S10. Coefficients from GLMER on Dipodomys energy use. 8

Table S11. Estimates from GLMER on Dipodomys energy use. 8

Table S12. Contrasts from GLMER on Dipodomys energy use. 9

C. baileyi proportional energy use 10

Model specification and selection 10

Table S13. Model comparison for C. baileyi proportional energy use. 10

Results 11

Table S14. Coefficients from GLMER on C. baileyi energy use 11
Table S15. Estimates from GLMER on C. baileyi energy use 11
Table S16. Contrasts from GLMER on C. baileyi energy use. 11
References 12

Explanation

In order to calculate energetic compensation and the total energy ratio, we require an estimate for the baseline values of total energy use, kangaroo rat energy use, and small granivore energy use on control plots. Estimating these baselines requires aggregating over between-plot variability among the control plots. For consistency, in the main analysis, we also aggregate across the exclosure plots and focus on treatment-level means throughout. Here, we explore the effect of between-plot variability on our analyses, to the extent possible. We used treatment-level means across control plots to calculate energetic compensation and the total energy ratio, but calculated these quantities separately for each exclosure plot, and conducted analyses including a random effect of plot. We also conducted analyses of *Dipodomys* and *C. baileyi* proportional energy use using plot-level data, again including plot as a random effect. Results were qualitatively the same as using treatment-level means.

Compensation

Model specification and selection

We fit linear mixed-effects models (using the lme function in the R package nlme; Pinheiro et al. 2021) of the form compensation ~ time period with a random effect of plot and temporal autocorrelation structure to account for autocorrelation between monthly census periods within each time period. We compared these to models without the autocorrelation structure, without the random effect, and without the term for time period. The best-fitting model included terms for time period, random effect of plot, and autocorrelation.

Table S1. Model comparison for compensation.

Model.specification	AIC
intercept + timeperiod + plot (random effect) + autocorrelation	1360.207
intercept + timeperiod + plot (random effect)	1680.916
intercept + timeperiod + autocorrelation	1409.830
intercept + plot (random effect) + autocorrelation	1408.362
intercept + plot (random effect)	1879.126
intercept	2036.371

Results

Table S2. Coefficients from linear mixed-effects model for compensation

Note that “oera” is the variable name for the term for time period in these analyses.

	Value	Std.Error	DF	t-value	p-value
(Intercept)	0.3451282	0.1048354	1362	3.292096	0.0010199
oera.L	0.0653090	0.0373313	1362	1.749446	0.0804392
oera.Q	-0.2845830	0.0341063	1362	-8.343990	0.0000000

Table S3. Estimates from linear mixed-effects model for compensation

Timeperiod	emmean	SE	df	lower.CL	upper.CL
1988-1997	0.1827673	0.1091842	3	-0.1647055	0.5302400
1997-2010	0.5774892	0.1078860	3	0.2341478	0.9208306

2010-2020	0.2751282	0.1093969	3	-0.0730215	0.6232779

Table S4. Contrasts from linear mixed-effects model for compensation

Comparison	estimate	SE	df	t.ratio	p.value
1988-1997 - 1997-2010	-0.3947220	0.0491845	1362	-8.025330	0.0000
1988-1997 - 2010-2020	-0.0923609	0.0527944	1362	-1.749446	0.1873
1997-2010 - 2010-2020	0.3023610	0.0496411	1362	6.090948	0.0000

Total energy use

Model specification and selection

As for compensation, we fit linear mixed-effects models fitting *total_energy_ratio ~ time period* with a random effect of plot and a temporal autocorrelation term to account for autocorrelation between monthly census periods within each timeperiod. We compared these to models without the autocorrelation term, without the random effect, and without the term for time period. The best-fitting model included terms for time period, random effect of plot, and autocorrelation.

Table S5. Model comparison for total energy ratio.

Model.specification	AIC
intercept + timeperiod + plot (random effect) + autocorrelation	474.8558
intercept + timeperiod + plot (random effect)	924.1830
intercept + timeperiod + autocorrelation	507.7842
intercept + plot (random effect) + autocorrelation	543.5425
intercept + plot (random effect)	1266.2097
intercept	1382.7469

Results

Table S6. Coefficients from linear mixed-effects model on total energy ratio

Note that "oera" is the variable name for the term for time period in these analyses.

	Value	Std.Error	DF	t-value	p-value
(Intercept)	0.5018200	0.0709701	1362	7.070865	0.0e+00
oera.L	0.1454309	0.0301324	1362	4.826392	1.5e-06
oera.Q	-0.2545852	0.0273660	1362	-9.302977	0.0e+00

Table S7. Estimates from linear mixed-effects model on total energy ratio

Timeperiod	emmean	SE	df	lower.CL	upper.CL
1988-1997	0.2950508	0.0751321	3	0.0559470	0.5341547
1997-2010	0.7096879	0.0738511	3	0.4746606	0.9447151

2010-2020	0.5007212	0.0752881	3	0.2611207	0.7403216

Table S8. Contrasts from linear mixed-effects model on total energy ratio

Comparison	estimate	SE	df	t.ratio	p.value
1988-1997 - 1997-2010	-0.4146370	0.0395736	1362	-10.477622	0.0e+00
1988-1997 - 2010-2020	-0.2056703	0.0426137	1362	-4.826392	4.6e-06
1997-2010 - 2010-2020	0.2089667	0.0398571	1362	5.242901	5.0e-07

Kangaroo rat proportional energy use

Model specification and selection

To compare proportional energy use across time periods, we used binomial generalized linear mixed models (using the glmer function in the R package lme4; Bates et al. 2015), which allowed us to include a random effect of plot.

For *Dipodomys* proportional energy use, we compared models with and without the random effect of plot and with and without a term for timeperiod. The best-fitting model included terms for timeperiod and a random effect of plot.

Table S9. Model comparison for Dipodomys proportional energy use.

Model.specification	AIC
intercept + timeperiod + plot (random effect)	1040.861
intercept + plot (random effect)	1162.470
intercept + timeperiod	1108.490
intercept	1208.081

Results

Table S10. Coefficients from GLMER on Dipodomys energy use.

Note that "oera" is the variable name for the term for time period in these analyses.

	Estimate	Std. Error	z value	Pr(>|z|)
(Intercept)	2.181163	0.1305753	16.704251	0
oera.L	-1.946096	0.2664545	-7.303670	0
oera.Q	1.124620	0.1769225	6.356572	0

Table S11. Estimates from GLMER on Dipodomys energy use.

Note that estimates are back-transformed onto the response scale, for interpretability.

Timeperiod	prob	SE	df	asymp.LCL	asymp.UCL
1988-1997	0.9823009	0.0062020	Inf	0.9701452	0.9944566
1997-2010	0.7795273	0.0183934	Inf	0.7434769	0.8155777
2010-2020	0.7797464	0.0208516	Inf	0.7388780	0.8206149

Table S12. Contrasts from GLMER on Dipodomys energy use.

Contrasts are performed on the link (logit) scale.

Comparison	estimate	SE	df	z.ratio	p.value
1988-1997 - 1997-2010	0.2027736	0.0194108	Inf	10.4464200	0
1988-1997 - 2010-2020	0.2025545	0.0217545	Inf	9.3109407	0
1997-2010 - 2010-2020	-0.0002191	0.0278048	Inf	-0.0078811	1

C. baileyi proportional energy use

Model specification and selection

As for kangaroo rat proportional energy use, we used a binomial generalized linear mixed effects model to compare *C. baileyi* proportional energy use across time periods. Because *C. baileyi* occurs on both control and exclosure plots, we investigated whether the dynamics of *C. baileyi*'s proportional energy use differed between treatment types. We compared models incorporating separate slopes, separate intercepts, or no terms for treatment modulating the change in *C. baileyi* proportional energy use across time periods, i.e. comparing the full set of models:

- *cbaileyi_proportional_energy_use ~ timeperiod + treatment + timeperiod:treatment*
- *cbaileyi_proportional_energy_use ~ timeperiod + treatment*
- *cbaileyi_proportional_energy_use ~ timeperiod*

We also tested a null (intercept-only) model of no change across time periods:

- *cbaileyi_proportional_energy_use ~ 1*

We compared all of these models with and without a random effect of plot.

We found that the best-fitting model incorporated a random effect of plot, and fixed effects for time period and for treatment, but no interaction between them (*cbaileyi_proportional_energy_use ~ timeperiod + treatment*). We therefore proceeded with this model.

Table S13. Model comparison for C. baileyi proportional energy use.

Model.specification	AIC
intercept + timeperiod + treatment + timeperiod:treatment + plot (random effect)	1021.318
intercept + timeperiod + treatment + plot (random effect)	1020.263
intercept + timeperiod + plot (random effect)	1042.758
intercept + plot (random effect)	1321.149
intercept + timeperiod + treatment + timeperiod:treatment	1166.653
intercept + timeperiod + treatment	1162.901
intercept + timeperiod	1869.097
intercept	2036.489

Results

Table S14. Coefficients from GLMER on C. baileyi energy use

Note that "oera" is the variable name for the term for time period in these analyses, and "oplottype" refers to experimental treatment.

	Estimate	Std. Error	z value	Pr(>|z|)
(Intercept)	-2.443643	0.2067789	-11.81766	0
oera.L	-1.866286	0.1530068	-12.19740	0
oplottype.L	3.265183	0.2913472	11.20719	0

Table S15. Estimates from GLMER on C. baileyi energy use

Note that estimates are back-transformed onto the response scale, for interpretability.

Timeperiod	Treatment	prob	SE	df	asymp.LCL	asymp.UCL
1997-2010	Control	0.0312856	0.0116044	Inf	0.0085414	0.0540297
1997-2010	Exclosure	0.7658194	0.0392864	Inf	0.6888195	0.8428193
2010-2020	Control	0.0023009	0.0008486	Inf	0.0006378	0.0039641
2010-2020	Exclosure	0.1893142	0.0364430	Inf	0.1178872	0.2607412

Table S16. Contrasts from GLMER on C. baileyi energy use.

Contrasts are performed on the link (logit) scale.

Comparison	Treatment	estimate	SE	df	z.ratio	p.value
1997-2010 - 2010-2020	Control	2.639326	0.2163843	Inf	12.1974	0
1997-2010 - 2010-2020	Exclosure	2.639326	0.2163843	Inf	12.1974	0

References

Bates, Douglas, Martin Maechler, Ben Bolker, Steve Walker (2015). *Fitting Linear Mixed-Effects Models Using lme4.* Journal of Statistical Software, 67(1), 1-48. doi:10.18637/jss.v067.i01.

Lenth, Russell V. (2021). emmeans: *Estimated Marginal Means, aka Least-Squares Means.* R package version 1.7.0. <URL: https://CRAN.R-project.org/package=emmeans>

Pinheiro J, Bates D, DebRoy S, Sarkar D, R Core Team (2021). *nlme: Linear and Nonlinear Mixed Effects Models*. R package version 3.1-153, <URL: https://CRAN.R-project.org/package=nlme>.
