# Supplementary material for: Maintenance of community function through compensation breaks down over time in a desert rodent community
Source: Ecology. 2022 May 12;103(7):e3709. doi: 10.1002/ecy.3709 (PMC9287087; doi:10.1002/ecy.3709)
Supplement: Supplementary file 2 — Appendix S2 [file ECY-103-0-s001.pdf]

Appendix S2 - Full analytical methods and model results

Supplemental information for "Maintenance of community function through compensation breaks down over time in a desert rodent community", by Renata M. Diaz and S. K. Morgan Ernest, in *Ecology.*

Fully annotated code and RMarkdown documents to reproduce these analyses are available at https://doi.org/10.5281/zenodo.5544361 and https://doi.org/10.5281/zenodo.5539880.

Table of Contents

Compensation 2

Table S1. Model comparison for compensation 2

Table S2. Coefficients from GLS for compensation 2

Table S3. Estimates from GLS for compensation 2

Table S4. Contrasts from GLS for compensation 3

Total energy use ratio 4

Table S5. Model comparison for total energy ratio. 4

Table S6. Coefficients from GLS on total energy ratio 4

Table S7. Estimates from GLS on total energy ratio 4

Table S8. Contrasts from GLS on total energy ratio 4

Kangaroo rat (Dipodomys) proportional energy use 6

Table S9. Model comparison for Dipodomys proportional energy use. 6

Table S10. Coefficients from GLM on Dipodomys energy use. 6

Table S11. Estimates from GLM on Dipodomys energy use. 6

Table S12. Contrasts from GLM on Dipodomys energy use. 6

C. baileyi proportional energy use 8

Table S13. Model comparison for C. baileyi proportional energy use. 8

Table S14. Coefficients from GLM on C. baileyi energy use 8

Table S15. Estimates from GLM on C. baileyi energy use 9

Table S16. Contrasts from GLM on C. baileyi energy use. 9

References 10

Compensation

We fit a generalized least squares (of the form *compensation ~ timeperiod*; note that "timeperiod" is coded as "oera" throughout) using the gls function from the R package nlme (Pinheiro et al. 2021). Because values from monthly censuses within each time period are subject to temporal autocorrelation, we included a continuous autoregressive temporal autocorrelation structure of order 1 (using the CORCAR1 function). We compared this model to models fit without the autocorrelation structure and without the time period term using AIC. The model with both the time period term and the autocorrelation structure was the best-fitting model via AIC, and we used this model to calculate estimates and contrasts using the package emmeans (Lenth 2021).

Table S1. Model comparison for compensation.

Model.specification	AIC
intercept + timeperiod + autocorrelation	69.85023
intercept + autocorrelation	84.74902
intercept + timeperiod	157.09726
intercept	252.74534

Table S2. Coefficients from GLS for compensation

Note that "oera" is the variable name for the term for time period in these analyses.

	Value	Std.Error	t-value	p-value
(Intercept)	0.3450313	0.0294996	11.696141	0.0000000
oera.L	0.0647933	0.0524103	1.236269	0.2172146
oera.Q	-0.2833553	0.0477359	-5.935890	0.0000000

Table S3. Estimates from GLS for compensation

Timeperiod	emmean	SE	df	lower.CL	upper.CL
1988-1997	0.1835362	0.0520378	44.11081	0.0786683	0.2884041
1997-2010	0.5763899	0.0462641	47.37851	0.4833383	0.6694416
2010-2020	0.2751677	0.0528010	46.75897	0.1689314	0.3814041

Table S4. Contrasts from GLS for compensation

Comparison	estimate	SE	df	t.ratio	p.value
1988-1997 - 1997-2010	-0.3928537	0.0689413	47.89422	-5.698378	0.0000
1988-1997 - 2010-2020	-0.0916315	0.0741194	45.51740	-1.236269	0.4383
1997-2010 - 2010-2020	0.3012222	0.0694989	49.52957	4.334200	0.0002

Total energy use ratio

As for compensation, we fit a generalized least squares of the form *total_energy_ratio ~ timeperiod*, accounting for temporal autocorrelation between monthly censuses within each time period using a continuous autoregressive autocorrelation structure of order 1. We compared this model to models fit without the timeperiod term and/or autocorrelation structure, and found the full (timeperiod plus autocorrelation) model had the best performance via AIC. We used this model for estimates and contrasts.

Table S5. Model comparison for total energy ratio.

Model.specification	AIC
intercept + timeperiod + autocorrelation	-132.92138
intercept + autocorrelation	-118.15000
intercept + timeperiod	13.29396
intercept	156.85988

Table S6. Coefficients from GLS on total energy ratio

Note that "oera" is the variable name for the term for time period in these analyses.

	Value	Std.Error	t-value	p-value
(Intercept)	0.5016731	0.0271176	18.499880	0.0000000
oera.L	0.1413504	0.0477646	2.959316	0.0033001
oera.Q	-0.2503659	0.0429312	-5.831790	0.0000000

Table S7. Estimates from GLS on total energy ratio

Timeperiod	emmean	SE	df	lower.CL	upper.CL
1988-1997	0.2995118	0.0475806	36.19943	0.2030323	0.3959913
1997-2010	0.7060960	0.0419773	38.51943	0.6211550	0.7910369
2010-2020	0.4994115	0.0480066	37.62774	0.4021956	0.5966274

Table S8. Contrasts from GLS on total energy ratio

Comparison	estimate	SE	df	t.ratio	p.value
1988-1997 - 1997-2010	-0.4065842	0.0623398	40.51631	-6.522060	0.0000

1988-1997 - 2010-2020	-0.1998997	0.0675493	37.12310	-2.959316	0.0144
1997-2010 - 2010-2020	0.2066845	0.0626456	41.44768	3.299267	0.0056

Kangaroo rat (Dipodomys) proportional energy use

Proportional energy use is bounded 0-1 and cannot be fit with generalized least squares. We therefore used a binomial generalized linear model of the form *dipodomys_proportional_energy_use ~ timeperiod*. We compared a model fit with a timeperiod term to an intercept-only (null) model using AIC, and found the timeperiod term improved model fit. We used this model for estimates and contrasts.

Note that we were unable to incorporate temporal autocorrelation into generalized linear models, and we prioritized fitting models of the appropirate family over accounting for autocorrelation. Due to the pronounced differences between time periods for these variables, we were comfortable proceeding without explicitly accounting for autocorrelation.

Table S9. Model comparison for Dipodomys proportional energy use.

Model.specification	AIC
intercept + timeperiod	258.3581
intercept	280.8497

Table S10. Coefficients from GLM on Dipodomys energy use.

Note that "oera" is the variable name for the term for time period in these analyses. Coefficients are given on the link (logit) scale.

	Estimate	Std. Error	z value	Pr(>|z|)
(Intercept)	1.4032480	0.1503201	9.335068	0.0000000
oera.L	-1.1000833	0.2871738	-3.830723	0.0001278
oera.Q	0.5855493	0.2304516	2.540878	0.0110574

Table S11. Estimates from GLM on Dipodomys energy use.

Note that estimates are back-transformed onto the response scale, for interpretability.

Timeperiod	prob	SE	df	asymp.LCL	asymp.UCL
1988-1997	0.9183528	0.0256462	Inf	0.8680872	0.9686183
1997-2010	0.7160901	0.0398537	Inf	0.6379782	0.7942020
2010-2020	0.7035835	0.0456677	Inf	0.6140765	0.7930905

Table S12. Contrasts from GLM on Dipodomys energy use.

Contrasts are performed on the link (logit) scale.

contrast	estimate	SE	df	z.ratio	p.value
a_pre_pb - b_pre_reorg	1.4950249	0.3942281	Inf	3.7922836	0.0004
a_pre_pb - c_post_reorg	1.5557527	0.4061251	Inf	3.8307227	0.0004
b_pre_reorg - c_post_reorg	0.0607279	0.2938992	Inf	0.2066282	0.9767

C. baileyi proportional energy use

As for kangaroo rat proportional energy use, we used a binomial generalized linear model to compare *C. baileyi* proportional energy use across time periods. Because *C. baileyi* occurs on both control and exclosure plots, we investigated whether the dynamics of *C. baileyi*'s proportional energy use differed between treatment types. We compared models incorporating separate slopes, separate intercepts, or no terms for treatment modulating the change in *C. baileyi* proportional energy use across time periods, i.e. comparing the full set of models:

- *cbaileyi_proportional_energy_use ~ timeperiod + treatment + timeperiod:treatment*
- *cbaileyi_proportional_energy_use ~ timeperiod + treatment*
- *cbaileyi_proportional_energy_use ~ timeperiod*

We also tested a null (intercept-only) model of no change across time periods:

- *cbaileyi_proportional_energy_use ~ 1*

We found that the best-fitting model incorporated effects for time period and for treatment, but no interaction between them (*cbaileyi_proportional_energy_use ~ timeperiod + treatment*). We therefore proceeded with this model.

Table S13. Model comparison for C. baileyi proportional energy use.

Model.specification	AIC
intercept + timeperiod + treatment + timeperiod:treatment	237.7643
intercept + timeperiod + treatment	231.0963
intercept + timeperiod	460.8477
intercept	541.3799

Table S14. Coefficients from GLM on C. baileyi energy use

Note that "oera" is the variable name for the term for time period in these analyses, and "oplottype" refers to treatment. Coefficients are given on the link (logit) scale.

	Estimate	Std. Error	z value	Pr(>|z|)
(Intercept)	-1.574028	0.1670168	-9.424368	0
oera.L	-1.409273	0.2010398	-7.009921	0
oplottype.L	2.184896	0.2267112	9.637355	0

Table S15. Estimates from GLM on C. baileyi energy use

Note that estimates are back-transformed onto the response scale, for interpretability.

Timeperiod	Treatment	prob	SE	df	asymp.LCL	asymp.UCL
1997-2010	Control	0.1069314	0.0258894	Inf	0.0561890	0.1576737
1997-2010	Exclosure	0.7246076	0.0385129	Inf	0.6491236	0.8000915
2010-2020	Control	0.0160560	0.0058224	Inf	0.0046444	0.0274676
2010-2020	Exclosure	0.2639419	0.0428458	Inf	0.1799657	0.3479181

Table S16. Contrasts from GLM on C. baileyi energy use.

Contrasts are performed on the link (logit) scale.

Comparison	Treatment	estimate	SE	df	z.ratio	p.value
1997-2010 - 2010-2020	Control	1.993013	0.2843132	Inf	7.009921	0
1997-2010 - 2010-2020	Exclosure	1.993013	0.2843132	Inf	7.009921	0

References

Lenth, Russell V. (2021). emmeans: *Estimated Marginal Means, aka Least-Squares Means.* R package version 1.7.0. <URL: https://CRAN.R-project.org/package=emmeans>

Pinheiro J, Bates D, DebRoy S, Sarkar D, R Core Team (2021). *nlme: Linear and Nonlinear Mixed Effects Models*. R package version 3.1-153, <URL: https://CRAN.R-project.org/package=nlme>.
