# Supplementary material for: Maintenance of community function through compensation breaks down over time in a desert rodent community
Source: Ecology. 2022 May 12;103(7):e3709. doi: 10.1002/ecy.3709 (PMC9287087; doi:10.1002/ecy.3709)
Supplement: Supplementary file 3 — Appendix S3 [file ECY-103-0-s003.pdf]

Appendix S3 - Biomass analysis

Supplemental information for "Maintenance of community function through compensation breaks down over time in a desert rodent community", by Renata M. Diaz and S. K. Morgan Ernest, in *Ecology*.

Fully annotated code and RMarkdown documents to reproduce these analyses are available at https://doi.org/10.5281/zenodo.5544361 and https://doi.org/10.5281/zenodo.5539880.

All statistical methods for biomass are identical to the ones for energy use (Appendix S1).

Table of Contents

Compensation 2
Table S1. Model comparison for compensation 2
Table S2. Coefficients from GLS for compensation 2
Table S3. Estimates from GLS for compensation 2
Table S4. Contrasts from GLS for compensation 3
Total biomass ratio 4
Table S5. Model comparison for total biomass ratio. 4
Table S6. Coefficients from GLS on total biomass ratio 4
Table S7. Estimates from GLS on total biomass ratio 4
Table S8. Contrasts from GLS on total biomass ratio 4
Kangaroo rat (Dipodomys) proportional biomass 6
Table S9. Model comparison for Dipodomys proportional biomass. 6
Table S10. Coefficients from GLM on Dipodomys biomass 6
Table S11. Estimates from GLM on Dipodomys biomass 6
Table S12. Contrasts from GLM on Dipodomys biomass. 6
C. baileyi proportional biomass 8
Model specification and selection 8
Table S13. Model comparison for C. baileyi proportional biomass. 8
Table S14. Coefficients from GLM on C. baileyi biomass 8
Table S15. Estimates from GLM on C. baileyi biomass 9
Table S16. Contrasts from GLM on C. baileyi biomass. 9
Figure S1. Biomass results 10
Figure S1 Legend. 11
References 12

Compensation

We fit a generalized least squares (of the form *compensation ~ timeperiod*; note that "timeperiod" is coded as "oera" throughout) using the gls function from the R package nlme (Pinheiro et al. 2021). Because values from monthly censuses within each time period are subject to temporal autocorrelation, we included a continuous autoregressive temporal autocorrelation structure of order 1 (using the CORCAR1 function). We compared this model to models fit without the autocorrelation structure and without the time period term using AIC. The model with both the time period term and the autocorrelation structure was the best-fitting model via AIC, and we used this model to calculate estimates and contrasts using the package emmeans (Lenth 2021).

Table S1. Model comparison for compensation.

Model.specification	AIC
intercept + timeperiod + autocorrelation	-17.623354
intercept + autocorrelation	-3.297103
intercept + timeperiod	92.184205
intercept	207.804481

Table S2. Coefficients from GLS for compensation

Note that "oera" is the variable name for the term for time period in these analyses.

	Value	Std.Error	t-value	p-value
(Intercept)	0.3081443	0.0290539	10.605950	0.0000000
oera.L	0.0711412	0.0514131	1.383719	0.1673549
oera.Q	-0.2799121	0.0465252	-6.016352	0.0000000

Table S3. Estimates from GLS for compensation

Timeperiod	emmean	SE	df	lower.CL	upper.CL
1988-1997	0.1435663	0.0511419	39.28312	0.0401458	0.2469867
1997-2010	0.5366915	0.0452745	41.91562	0.4453185	0.6280646
2010-2020	0.2441751	0.0517205	41.17937	0.1397373	0.3486130

Table S4. Contrasts from GLS for compensation

Comparison	estimate	SE	df	t.ratio	p.value
1988-1997 - 1997-2010	-0.3931253	0.0673811	43.22895	-5.834358	0.0000
1988-1997 - 2010-2020	-0.1006089	0.0727090	40.36882	-1.383719	0.3588
1997-2010 - 2010-2020	0.2925164	0.0678003	44.43055	4.314383	0.0003

Total biomass ratio

As for compensation, we fit a generalized least squares of the form *total_biomass_ratio ~ timeperiod*, accounting for temporal autocorrelation between monthly censuses within each time period using a continuous autoregressive autocorrelation structure of order 1. We compared this model to models fit without the timeperiod term and/or autocorrelation structure, and found the full (timeperiod plus autocorrelation) model had the best performance via AIC. We used this model for estimates and contrasts.

Table S5. Model comparison for total biomass ratio.

Model.specification	AIC
intercept + timeperiod + autocorrelation	-176.57761
intercept + autocorrelation	-162.61339
intercept + timeperiod	-15.98438
intercept	146.61442

Table S6. Coefficients from GLS on total biomass ratio

Note that "oera" is the variable name for the term for time period in these analyses.

	Value	Std.Error	t-value	p-value
(Intercept)	0.4553971	0.0272418	16.716827	0.0000000
oera.L	0.1454493	0.0477989	3.042941	0.0025257
oera.Q	-0.2531409	0.0427343	-5.923594	0.0000000

Table S7. Estimates from GLS on total biomass ratio

Timeperiod	emmean	SE	df	lower.CL	upper.CL
1988-1997	0.2492046	0.0476584	33.82432	0.1523326	0.3460765
1997-2010	0.6620857	0.0419515	35.98516	0.5770030	0.7471684
2010-2020	0.4549009	0.0480215	34.98703	0.3574107	0.5523911

Table S8. Contrasts from GLS on total biomass ratio

Comparison	estimate	SE	df	t.ratio	p.value
1988-1997 - 1997-2010	-0.4128811	0.0621739	38.42746	-6.640747	0.0000

1988-1997 - 2010-2020	-0.2056963	0.0675979	34.67694	-3.042941	0.0121
1997-2010 - 2010-2020	0.2071848	0.0624325	39.20390	3.318542	0.0054

Kangaroo rat (Dipodomys) proportional biomass

Proportional biomass is bounded 0-1 and cannot be fit with generalized least squares. We therefore used a binomial generalized linear model with no temporal autocorrelation term, of the form *dipodomys_proportional_biomass ~ timeperiod*. We compared a model fit with a timeperiod term to an intercept-only (null) model using AIC, and found the timeperiod term improved model fit. We used this model for estimates and contrasts.

Table S9. Model comparison for Dipodomys proportional biomass.

Model.specification	AIC
intercept + timeperiod	215.2069
intercept	227.9608

Table S10. Coefficients from GLM on Dipodomys biomass.

Note that "oera" is the variable name for the term for time period in these analyses. Coefficients are given on the link (logit) scale.

	Estimate	Std. Error	z value	Pr(>|z|)
(Intercept)	1.6149566	0.1644937	9.817741	0.0000000
oera.L	-1.1672395	0.3180813	-3.669626	0.0002429
oera.Q	0.6619048	0.2473324	2.676175	0.0074468

Table S11. Estimates from GLM on Dipodomys biomass.

Note that estimates are back-transformed onto the response scale, for interpretability.

Timeperiod	prob	SE	df	asymp.LCL	asymp.UCL
1988-1997	0.9376458	0.0226460	Inf	0.8932605	0.9820310
1997-2010	0.7454543	0.0385025	Inf	0.6699909	0.8209177
2010-2020	0.7426552	0.0437171	Inf	0.6569713	0.8283392

Table S12. Contrasts from GLM on Dipodomys biomass.

Contrasts are performed on the link (logit) scale.

contrast	estimate	SE	df	z.ratio	p.value

a_pre_pb - b_pre_reorg	1.6360275	0.4372643	Inf	3.741508	0.0005
a_pre_pb - c_post_reorg	1.6507259	0.4498349	Inf	3.669626	0.0007
b_pre_reorg - c_post_reorg	0.0146984	0.3057707	Inf	0.048070	0.9987

C. baileyi proportional biomass

Model specification and selection

As for kangaroo rat proportional biomass, we used a binomial generalized linear model to compare *C. baileyi* proportional biomass across time periods. Because *C. baileyi* occurs on both control and exclosure plots, we investigated whether the dynamics of *C. baileyi*'s proportional biomass differed between treatment types. We compared models incorporating separate slopes, separate intercepts, or no terms for treatment modulating the change in *C. baileyi* proportional biomass across time periods, i.e. comparing the full set of models:

- *cbaileyi_proportional_biomass ~ timeperiod + treatment + timeperiod:treatment*
- *cbaileyi_proportional_biomass ~ timeperiod + treatment*
- *cbaileyi_proportional_biomass ~ timeperiod*

We also tested a null (intercept-only) model of no change across time periods:

- *cbaileyi_proportional_biomass ~ 1*

We found that the best-fitting model incorporated effects for time period and for treatment, but no interaction between them (*cbaileyi_proportional_biomass ~ timeperiod + treatment*). We therefore proceeded with this model.

Table S13. Model comparison for C. baileyi proportional biomass.

Model.specification	AIC
intercept + timeperiod + treatment + timeperiod:treatment	237.6847
intercept + timeperiod + treatment	231.2374
intercept + timeperiod	466.4937
intercept + treatment	346.2154
intercept	543.7811

Table S14. Coefficients from GLM on C. baileyi biomass.

Note that "oera" is the variable name for the term for time period in these analyses, and "oplottype" refers to treatment. Coefficients are given on the link (logit) scale.

	Estimate	Std. Error	z value	Pr(>|z|)
(Intercept)	-1.538798	0.1671239	-9.207525	0
oera.L	-1.403286	0.2006948	-6.992140	0

oplottype.L	2.270657	0.2298594	9.878462	0

Table S15. Estimates from GLM on C. baileyi biomass

Note that estimates are back-transformed onto the response scale, for interpretability.

Timeperiod	Treatment	prob	SE	df	asymp.LCL	asymp.UCL
1997-2010	Control	0.1041331	0.0255800	Inf	0.0539971	0.1542691
1997-2010	Exclosure	0.7425132	0.0376727	Inf	0.6686761	0.8163504
2010-2020	Control	0.0157248	0.0057341	Inf	0.0044861	0.0269634
2010-2020	Exclosure	0.2838438	0.0439192	Inf	0.1977637	0.3699240

Table S16. Contrasts from GLM on C. baileyi biomass.

Contrasts are performed on the link (logit) scale.

Comparison	Treatment	estimate	SE	df	z.ratio	p.value
1997-2010 - 2010-2020	Control	1.984546	0.2838253	Inf	6.99214	0
1997-2010 - 2010-2020	Exclosure	1.984546	0.2838253	Inf	6.99214	0

Figure S1. Biomass results

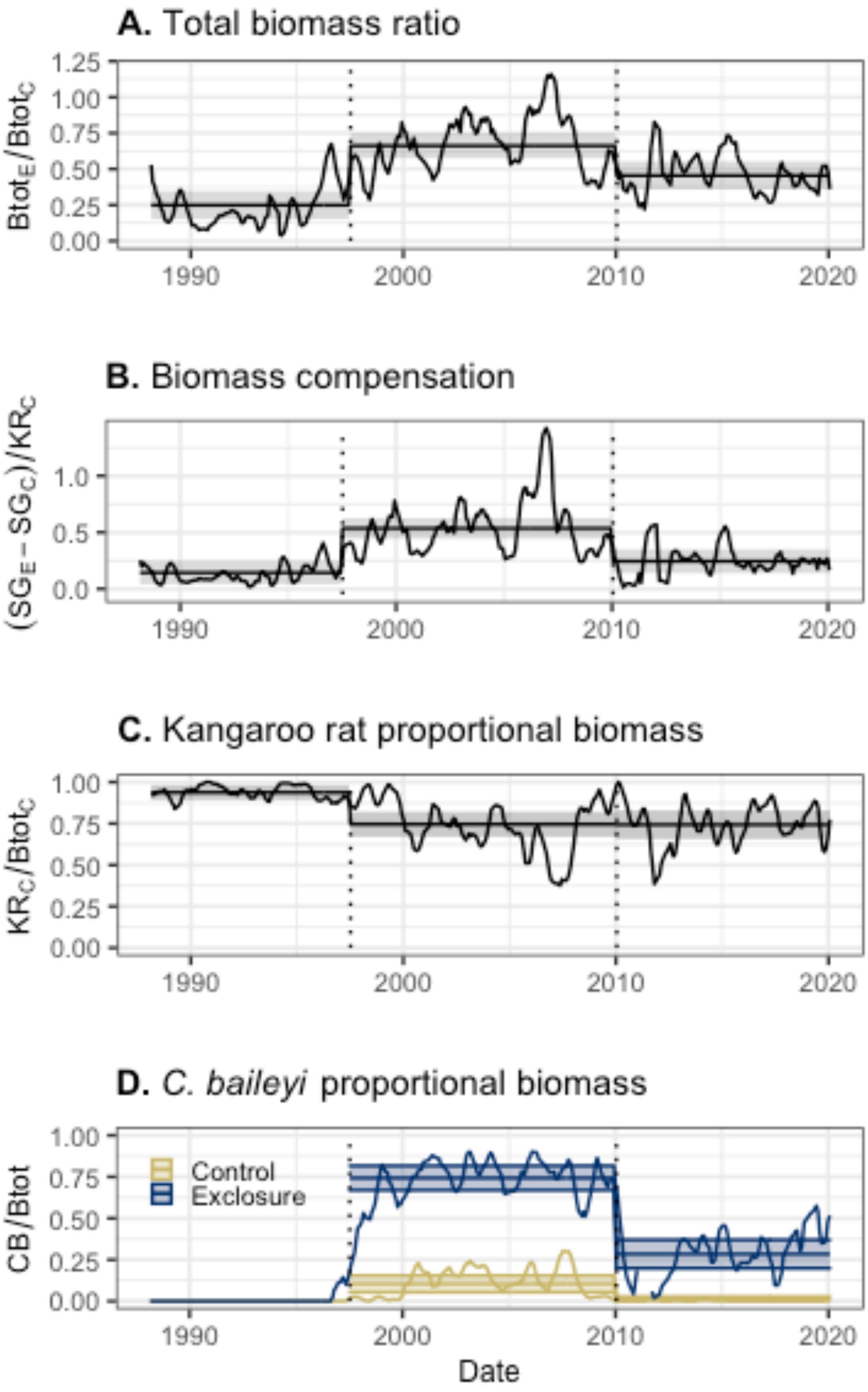

Figure S1 Legend.

Dynamics of biomass and rodent community composition over time. Lines represent the ratio of biomass on exclosure plots to control plots (a), 6-month moving averages of biomass compensation (b), and the share of community-wide biomass accounted for by kangaroo rats on control plots (c), and by C. baileyi (d), on control (gold) and exclosure (blue) plots. Dotted vertical lines mark the boundaries between time periods used for statistical analysis. Horizontal lines are time-period estimates from generalized least squares (a, b) and generalized linear (c, d) models, and the semitransparent envelopes mark the 95% confidence or credible intervals.

References

Lenth, Russell V. (2021). emmeans: *Estimated Marginal Means, aka Least-Squares Means.* R package version 1.7.0. <URL: https://CRAN.R-project.org/package=emmeans>

Pinheiro J, Bates D, DebRoy S, Sarkar D, R Core Team (2021). *nlme: Linear and Nonlinear Mixed Effects Models*. R package version 3.1-153, <URL: https://CRAN.R-project.org/package=nlme>.
